# Product Integration of Established Crash Sensors for Safety Applications in Lightweight Vehicles

**DOI:** 10.3390/s21216994

**Published:** 2021-10-21

**Authors:** Linda Klein, Yvonne Joseph, Matthias Kröger

**Affiliations:** 1 Robert Bosch GmbH, Powertrain Solutions, 70469 Stuttgart, Germany; 2 Institute of Electronic and Sensor Materials, Technische Universität Bergakademie Freiberg, 09599 Freiberg, Germany; Yvonne.Joseph@esm.tu-freiberg.de; 3 Institute for Machine Elements, Design and Manufacturing, Technische Universität Bergakademie Freiberg, 09599 Freiberg, Germany; Kroeger@imkf.tu-freiberg.de

**Keywords:** sensor integration, automotive sensors, safety applications, crash sensing, condition detection, lightweight vehicles, composites, fibre-reinforced polymers

## Abstract

The functionality of products increases when more sensors are used. This trend also affects future automobiles and becomes even more relevant in connected and autonomous applications. Concerning automotive lightweight design, carbon fibre-reinforced polymers (CFRP) are suitable materials. However, their drawbacks include the relatively high manufacturing costs of CFRP components in addition to the difficulty of recycling. To compensate for the increased expenditure, the integration of automotive sensors in CFRP vehicle structures provides added value. As a new approach, established sensors are integrated into fibre-reinforced polymer (FRP) structures. The sensors are usually mounted to the vehicle. The integration of sensors into the structure saves weight and space. Many other approaches specifically develop new sensors for integration into FRP structures. With the new approach, there is no need for elaborate development of new sensors since established sensors are used. The present research also showed that the range of applications of the sensors can be extended by the integration. The present paper outlines the functional behaviour of the integrated sensor utilized for crashing sensing. First of all, the integration quality of the sensor is relevant. Different requirements apply to the usual mounting of the sensor. The self-sensing structure must fulfil those requirements. Moreover, unfamiliar characteristics of the new surrounding structure might affect the sensing behaviour. Thus, the sensing behaviour of the self-sensing composite was analyzed in detail. The overarching objective is the general integration of sensors in products with reasonable effort.

## 1. Introduction

The functionality of vehicles, and products in general, increases and is often solved by sensor applications. Sensors become even more relevant in connected vehicles and autonomous applications (e.g., autonomous driving), especially for safety-relevant applications. A challenge is the number of sensors, especially when the installation space of the vehicle or the product is limited. Automotive applications where sensors are involved will increase in the future. Due to high demands on functional safety, the development effort for new sensors is time- and cost-intensive. In addition, the complexity of sensor systems raises and the logistics and assembly effort gets higher. One solution is the integration of sensors into the product. The combination of the sensor and the product enables a space-saving solution that shortens the process chain during production.

The present research deals with a topic in the field of sensor integration in fibre-based composite structures. As an application example, a new integration approach was demonstrated for an automotive sensor to be integrated into lightweight vehicle structures. Carbon fibre-reinforced polymers (CFRP) are suitable materials for these structures. Especially the high energy absorption makes CFRP well suited for energy-absorbing components, such as rocker panels or crash boxes. Then again, with respect to the sensor integration, the set-up in layers of fibre-reinforced polymer (FRP) structures has the potential to integrate crash sensors. The present research is a contribution to this.

## 2. Current State of Research

FRP structures are already established in the aircraft sector for the objective of lightweight design. Therefore, the development of sensors for integration in FRP structures has been promoted. Major applications of the integrated sensors are the measuring of loads, structural health monitoring or process monitoring during the manufacturing of a FRP structure. The developments of such sensors include piezoceramic transducers [1,2], piezoelectric wafer active sensors [3] and arrays of piezoceramic modules [4]. Similar integrable sensor technologies are nodes of ultrasonic transducers [5], piezo patches [6], fibre optical sensors [7], fibre bragg gratings [8,9] or optical silicone-based multimode fibres [10]. Additionally, chip-based resistors [11], silicon sensors [12,13] or phase array ultrasonic sensors [8] have been developed for an integration in FRP structures. Further developments are foil-based flexible sensors [12,14,15] or sensor layouts which are printed with conductive ink on textile fabrics. A similar example is integrable aluminium sheets with a film of thermoplastic melt filled with piezoceramic powder [16]. Furthermore, strain gauges were distributed over FRP structures to monitor mechanical loads [7,17]. A sensor as a part of a fibre grid fabric was realized by e.g., glass fibres with an electrically conductive sizing of carbon nano tubes (CNT) [18], piezoelectric fibres of polyvinylidene fluoride [19], strain-sensitive carbon fibres [20] and CNT yarns [21,22].

Other technologies for integrated sensing are the comparative vacuum monitoring (Structural Monitoring Systems Ltd, Perth, Australia) or buckypaper [23]. Beyond that, stretchable networks of pressure sensors and temperature sensors measured environmental loads inside a FRP structure [24]. Likewise, to detect humidity, polyimide foils with a sensitive dielectric were integrated [15]. In a composite coiled profile a combination of strain and temperature sensing was based on single mode optical fibres [25]. Other examples of sensor technologies, which were used for the process monitoring, are fibre-based flat electrodes (SMARTweave method) [26], grids of several dielectrical sensors [27], fringing electric field sensors [26] or also buckypaper [23]. Further, micro-thermocouples [26,28], fibre-optical refractometers [9,26,29], interferometers [26,29,30] or spectrometers [26,29,31], direct current resistance sensors [31], conductive filaments [30] or micromeshes [26] were used.

This overview gives an impression of the variety of sensor technologies that have been considered for the integration in FRP structures. Predominantly, integrable sensors were specially developed on this basis. The main application areas of the self-sensing structures are aviation, astronautics, mechanical engineering, robotics, wind energy and offshore; the automotive sector scarcely appears. In addition, the implementation of (automotive) crash sensing by self-sensing structures has not yet been in the focus of the current state of research. Automotive sensors, inter alia for the crash sensing, are usually bolted to the (metallic) vehicle body. Regarding FRP a bolt connection is a non-fibre-fair design element. Therefore, fibre-fair joining technologies have also been a major field of employment (i.a. [32,33,34,35,36]). A number of research projects deal with the character of joints for FRP structures under different loads [33,37,38,39,40].

## 3. Motive and Aim

Unlike many other approaches, within the present research, a sensor was not specifically developed for integration. Instead, an automotive sensor as a serial product was integrated into FRP structures. The new approach derives the following benefits for future lightweight vehicles. They provide a technological added value that should compensate the increased expenditure of CFRP vehicle structures:

- The transfer of established sensors is possible for future FRP vehicle structures. There is no need to develop and validate new sensors or sensor joints.
- The number of independently mounted sensors, as well as the wiring harness system, is reduced, additionally saving weight and construction space of a vehicle.
- Several sensing applications are well combinable; mounted sensors, connectors and the cable harness limit that. Automotive sensor concepts can be more complex.
- The integration opens up the potential of additional sensor applications for established sensors. This extends the application range of these sensors.

New applications within the present research were the use of the sensor for the condition detection of the surrounding structure (detailed description in [41,42,43]) and the process monitoring during the production of the structure [44,45,46,47,48,49,50,51,52,53].

## 4. Technological Implementation

**Figure 1:**
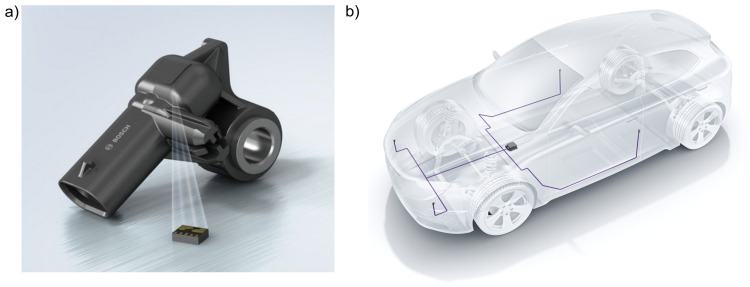
Automotive acceleration sensor: (**a**) sensor assembly, (**b**) vehicle mounting sites.

The used automotive sensor was an acceleration sensor as part of the automotive crash sensing. Within a measurement range of ±120 g, the sensor provides information on the direction and the level of an impact. The sensor is usually bolted upfront and peripheral to the vehicle body (Figure 1b). Additional pressure sensors are part of the crash sensing concept. The control unit is located central to the vehicle. Acceleration sensors also equip it.

The acceleration sensor’s overall weight is 10.5 g with dimensions of (*L* × *W* × *H*) 40 mm × 25 mm × 10 mm. It is an assembly of a housing, a connector interface, an over-moulded bolt plus the sensor module and its contacting on the inside of the housing (Figure 1a). The sensor module is a micro electro mechanical system (MEMS), weight 0.08 g and dimensions of (*L* × *W* × *H*) 4 mm × 5 mm × 1.6 mm. Inside the sensor module is the sensor element, which consists of interdependent micromechanical comb structures with electrodes on which seismic masses are hung up. Once an acceleration acts on the vehicle, a relative movement of the seismic masses leads to a quantitatively measurable capacity change, which is converted to a voltage. A voltage interface transfers the measured signal to the control unit [54,55]. The electric interface and the data protocol of the sensor correspond to the peripheral sensor interface 5 (PSI5). PSI5 is a universal interface specification for two-wire contacting. It is used for various automotive sensors [55].

**Figure 2:**
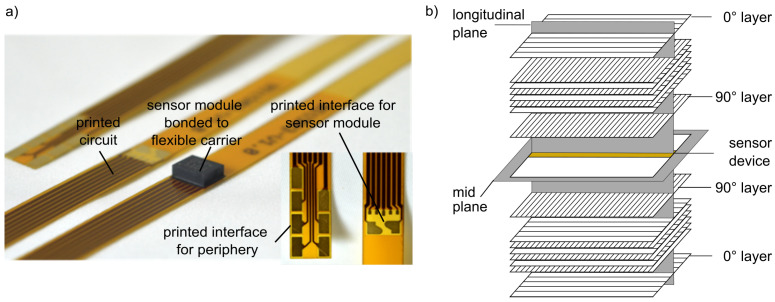
Sensor integration: (**a**) sensor device [43], (**b**) laminate lay-up, variant 2.

The approach for the integration into FRP structures only used the sensor module of the sensor. New electrical contacts were developed consisting of a flexible circuit carrier (Figure 2a, detailed description in [41,42,43]) [56,57]; in the following, the new sensor packaging is denoted as the sensor device. The measuring principal of the sensor was not changed for the sensor device. The design of the sensor device uses state of the art electronics. An automated reel-to-reel process can realize the assembling of the sensor device for future production of larger series.

The sensor device has different advantages for the target application of lightweight vehicles. Firstly, on the flex carrier, the sensor module is orientated and fixed precisely within the vehicle structure. Secondly, the flexible carrier can follow various shapes, which gives geometrical flexibility for self-sensing composites. Thirdly, the necessary construction space does not significantly increase with a rising number of sensors. The size of the sensor device is clearly reduced compared to the established automotive sensor. In addition, the bonding of a number of sensor modules is possible on only one flexible carrier, which reduces the amount of individual contacting.

The manufacturing process of the self-sensing composite was the resin transfer moulding (RTM). The RTM is applicable for serial production. In addition, it is a common technology based on liquid composite moulding (LCM). Therefore, the manufacturing process is transferable to different LCM technologies. Comprehensive process studies served to develop a tooling technology (detailed description in [58,59,60,61,62]) [44,45,63,64,65].

The experimental components of the present experiments were 4 mm thick structural plates. They consist of eight layers of carbon fibre grid fabric in a symmetrical laminate lay-up. The sensor device is located in the midplane of the stack (Figure 2b). Two different structure variants were prepared: variant 1 consists of fibres, which are orientated ±45° to the integrated sensor device, variant 2 has a fibre orientation of 0°/90° to the device. The matrix material was an epoxy resin (thermoset, detailed description of materials and manufacturing in [62]).

**Figure 3:**
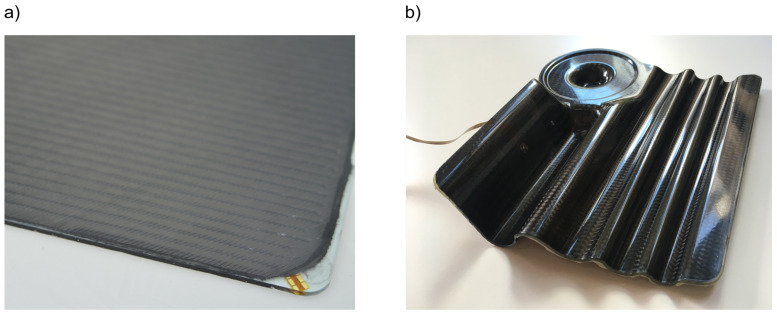
Self-sensing CFRP composite: (**a**) structural plate [41], (**b**) complex geometry.

The structure design of the present integration approach included a concealed installation of the sensor. The structure has two-sided smooth surfaces excluding elevations even for complex geometries (Figure 3).

## 5. Structural Component Quality and Mechanics

Concerning the adequate quality of the self-sensing composite, two aspects are relevant. Firstly, the typical criterion that determine the character of fibre-reinforced thermoset structures: the degree of cross-linking of the resin, the fibre content, the fibre impregnation and the fibre-matrix-adhesion. Secondly, the integration quality, which relates to the condition locally at the integrated sensor module: the position and the capsulation of the sensor module inside the FRP structure and the fibre deflection at the material inclusion (sensor module). For faultless sensing, the integration quality is essential. It links to the functional behaviour of the self-sensing composite also for the crash sensing. On that account, integration quality is one major topic of this paper. Additionally, a summary of the mechanical properties is given. A comprehensive discussion on the overall component quality along with the mechanical behaviour and the failure modes of the self-sensing structure will be published in [62].

### 5.1. Methods and Materials

For the analysis of the overall structural component quality thermal and chemical test methods were used in combination with optical tests (computer tomography (CT), scanning electron microscope, energy dispersive X-ray). To evaluate the integration quality served the scans of a computer tomograph Vltomelx with a microfocus tube of 225 kV (GE Sensing and Inspection Technologies, Pforzheim, Germany). Mechanical tests under static loads according to DIN standard served to evaluate the mechanical behaviour. The test specimens were conditioned to ambient conditions in accordance with DIN EN 2743 before the mechanical tests. The failure mode of the composites was analyzed by CT.

### 5.2. Results

#### 5.2.1. Integration Quality

**Figure 4:**
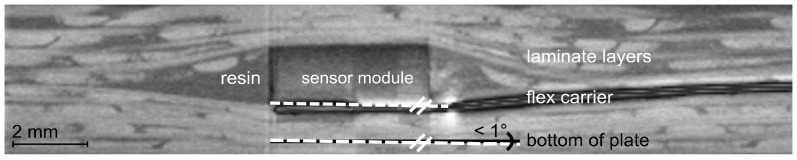
Position of the sensor module inside the structure [0/90]_8_ (computertomography).

For the valuation the position of the sensor module inside the structure, the electrically contacted lower surface of the sensor module was referenced to the bottom of the structural plate. The measured angular deviation was less than 1° for both structure variants. Figure 4 shows a cross-section of the self-sensing composite [0/90]_8_ (variant 2) at the location of the sensor module. The angular deviation between the sensor module and the bottom of the structural plate is marked in the scan.

**Figure 5:**
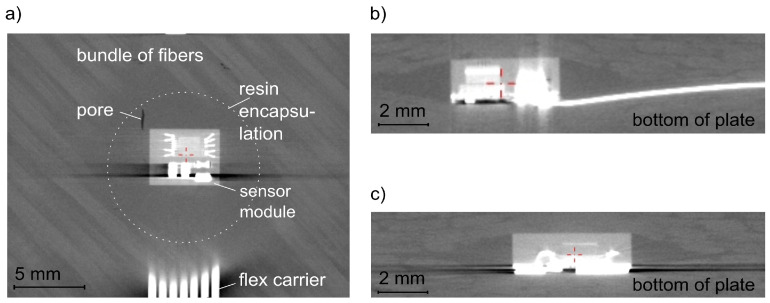
Integration quality at the sensor module: (**a**) top view, (**b**) side view, (**c**) frontal view (computertomography).

Figure 4 also shows that the flex carrier is bonded to the FRP structure without undulation. In CT scans, which were taken after applied mechanical loads to the structure (compression, tension, bending), the carrier still indicated that bonding to the fragments of the broken test specimens. The sensor module in Figure 4 is completely encapsulated by the resin. Lateral to the module are no resin-free areas. The resin and the boarding laminate layers identically mould the shape of the module. Additionally representative are the scans in Figure 5 of the top view (a), and the scans of the cross-section (b), (c) of the self-sensing composite [±45]_8_ (variant 1). They show the good quality of the resin encapsulation. The encapsulation has no defects or cracks and is almost free of pores. The size and the number of pores indicated that the present porosity was uncritical. It was comparable to a conventional epoxy resin cast of electronics [66]. The dimension of the circular encapsulation was measured within the CT scans. In Figure 5a it is approximately marked. The average diagonal of the area was 10.2 mm.

**Figure 6:**
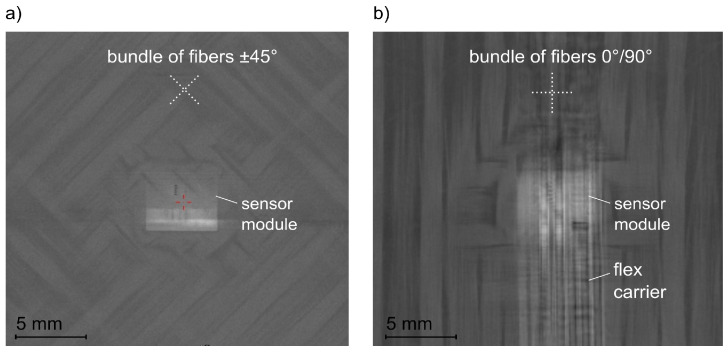
Deflection of fibres at the sensor module: (**a**) structure [±45]_8_, (**b**) structure [0/90]_8_ (computertomography).

Figure 6 represents the fibre deflection at the sensor module inside the structure: the CT scan of the top view of the structure (a) [±45]_8_ (variant 1) and (b) [0/90]_8_ (variant 2). Both scans show an undisturbed path of the fibres along the sensor module. The integration design with a concealed installation of the sensor makes the misalignment of the fibres above and below the sensor module unavoidable (Figure 4 and Figure 5b,c). However, the scans of the top view (Figure 6) show that the fibres did not also deflect laterally.

#### 5.2.2. Mechanical Properties

The mechanical specific values in Table 1 demonstrate the mechanical behaviour of both structure variants (±45° and 0°/90°). The self-sensing composite was compared to a *regular composite* and a *reference composite*. The *regular composite* had an identical laminate lay-up and the same plate thickness of 4 mm but no integrated sensor device. The *reference composite* had an identical lay-up with the maximum number of textile layers (thickness 4 mm); equally, it had no integrated sensor device.

In Table 1 significant differences between the specific mechanical values are indicated: for the comparison self-sensing/reference composite in column 3, for the comparison self-sensing/regular structure in column 5. The rating is (*) for much evidence to reject the null hypothesis, (**), for very much evidence to reject the null hypothesis and [***] if almost everything suggests rejecting the null hypothesis [67]. The null hypothesis claims that there are no effects.

The integration design with a concealed installation of the sensor limits the achievable fibre content of the self-sensing composite. It was only 60% of the reference composite’s fibre content. Direct effects were apparent in the tensile modulus. The modulus of the self-sensing composite reduced in the same ratio as the fibre contents. The reduction corresponds to the known behaviour of FRP structures. Their stiffness is predominantly determined by the fibres and thus by the fibre content [68]. The comparison of the self-sensing composite with the regular composite did not show a statistically significant difference in the tensile moduli. For the self-sensing structure, the integrated sensor device, therefore, did not directly influence the tensile modulus. However, a positive influence on the modulus by the integrated device was noticed under compression load for the composite [±45]_8_. The compression modulus of the self-sensing composite [±45]_8_ was significantly increased compared with the regular composite. The main cause was the laminate lay-up. As the fibres were oriented not directly but at 45° to the applied load, they did not withstand the compressive forces. Only the sensor device was oriented in the load direction. The device, therefore, absorbed parts of the compressive forces. In addition, a positive effect by the integrated sensor device was observed in the range of large strains during tensile load. The self-sensing composite [0/90]_8_ showed a significant increase in the tensile strength and in the elongation at break compared with the regular composite.

The effects due to the integrated sensor devices can be traced back to the low fibre content of the self-sensing structure. It might be reasonably assumed that the effect will decline with an increased fibre content of the self-sensing structure.

**Figure 7:**
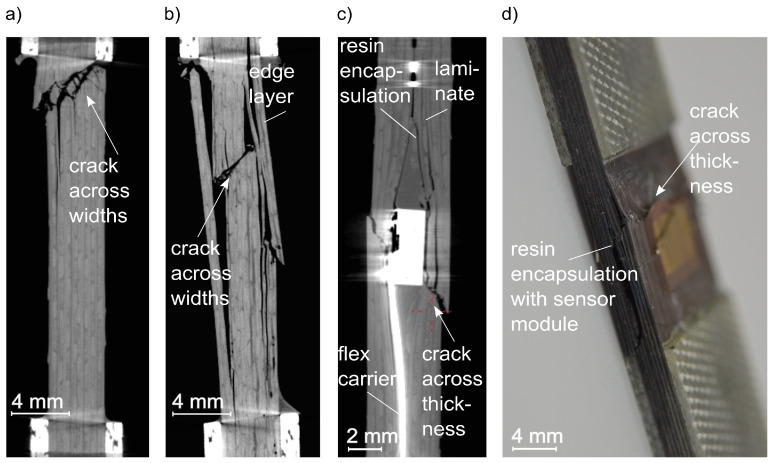
Failure after the compression test [0/90]_S_: (**a**) regular composite, (**b**) reference composite, (**c**,**d**) self-sensing composite (computertomography).

As an example, Figure 7 represents the position of failure of the composites [0/90]_S_ after the compression test (further comprehensive discussion on the failure modes of the self-sensing structure will be published in [62]). The integrated sensor device was the weakness of the self-sensing composite, which led to structural failure under tensile and compression load. The root cause was the encapsulated sensor module on the sensor device. A misalignment of the fibres encourages the buckling of fibres under a compressive load. In addition, the resin encapsulation of the sensor module has the character of an inclusion inside the structure. Inclusions cause peak stresses and delamination, which lead to cracks.

It is interesting that after the tensile and the compression load the adhesion of the flex carrier inside the composite persisted even after the structural failure. The adhesive strength between the flex carrier and the enclosing laminate layers seemed to dominate against the shear forces caused by the stresses.

Only after the bending load, the integrated sensor device had no obvious effect on the mechanics or the failure behaviour of the self-sensing composite. The structural failure of the self-sensing composite corresponded to the usual behaviour of a (regular) FRP structure.

### 5.3. Discussion

The integration quality of the self-sensing composite was examined by relevant criteria which can possibly influence the sensing behaviour. The integration quality met the requirements for the correct sensing of the established sensor. For the established mounting of the automotive acceleration sensor to the vehicle structure, a maximum permissible angular deviation is required by the sensor specification. The integrated sensor device did not exceed this value inside the structure.

The examination results also showed that the resin completely encapsulated the sensor module. The encapsulation is relevant for a rigid connection of the sensor module inside the structure. Further analysis of the interfaces between the resin encapsulation and the boarding laminate layers is appropriate, to support that finding.

The encapsulation has a good quality. Thus, the sensor module is insulated from the conductive carbon fibres of the FRP structure. This is important to protect the electronics of the sensor module from penetrating media and a corrosive attack. The quality and the rigid connection of the sensor module inside the structure are also mandatory for a sufficient force transmission into the FRP structure. Additionally, the mechanical behaviour is influenced by the orientation and deflections of the fibres [68]. For the present structure design, a fibre misalignment at the sensor module was a premise of the concealed installation. However, the results showed that the misalignment was held to a minimum.

The conclusion is that the integration of an established automotive sensor in an FRP structure is possible. Interference of the sensing functionality due to an inferior structural quality was avoided by an adjusted design and an adequate manufacturing technology of the self-sensing composite.

Finally, the low fibre content limits the use of the self-sensing composite because it greatly reduces the mechanical performance. This has to be taken into account for the application site of a vehicle structure. What should be sought is a higher technologically appropriate fibre content for the self-sensing structure. This should be achieved in the future by a thinning of the sensor module. The enclosure inside the self-sensing structure will be reduced and might prevent early failure.

Overall, the static mechanical tests should be supplemented by mechanical dynamic tests and impact tests. The fatigue life of the self-sensing structure might be reduced due to the fibre deflection at the integrated sensor module. The structural behaviour under thermal loads and under the influence of media is also relevant. Reliability analyses over the entire component life cycle are therefore recommended. In particular, the interface between the integrated sensor device and the surrounding laminate layers should be investigated. In the case of a load, interlaminar stress concentrations can lead to stress redistribution, which affects the structural mechanics.

## 6. Crash Sensing

Prior to the analysis of the crash sensing was an evaluation of the self-sensing composite’s principle sensing behaviour. The evaluation included the structure’s behaviour in new conditions and after environmental loads were applied.

On this basis, the crash sensing was analyzed by evaluating the principle functional behaviour of the self-sensing composite during a collision.

### 6.1. Methods and Materials

**Figure 8:**
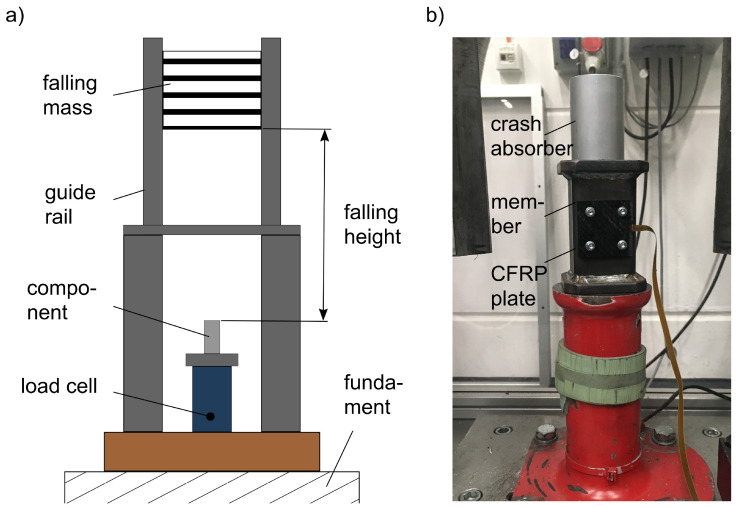
Test set-up of component test: (**a**) drop tower, (**b**) component with self-sensing composite.

The testing method for the functional behaviour of the self-sensing composite during a collision was a component test in a drop tower (Figure 8a). The component was a metallic side member to which the self-sensing composite was rigidly mounted (Figure 8b). Thus, the side member and the self-sensing composite plate formed an entity. The component was combined with an aluminium crash absorber. Crash absorbers are metallic deformation elements which, in combination with a side member, dissipate parts of the kinetic energy during a vehicle collision [69,70].

**Figure 9:**
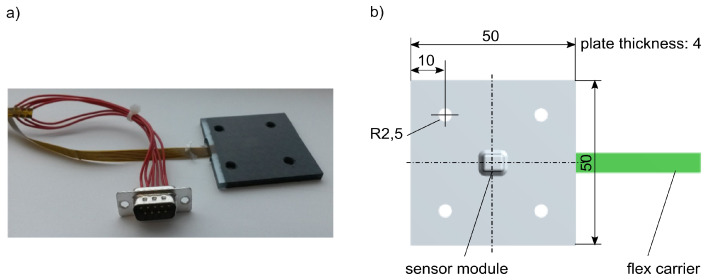
Test plate: (**a**) self-sensing composite with connector [73] (**b**) dimensions in mm.

The aluminium absorber was a tube of (*D* × *H*) 50 mm × 70 mm, wall thickness 2 mm. The crushing behaviour of its deformation during a collision was already known and reproducible [69,71,72]. The self-sensing composite was a CFRP planar plate of (*L* × *W* × *H*) 50 mm × 50 mm × 4 mm (Figure 9). Four drill holes served for the fixing of the plate to the side member. The plate had one integrated sensor device with the sensor module located at the centre of the plate. One end of the sensor device was fed out of the side of the plate. A PSI5 Simulyzer USB box (SesKion, Leinfelden-Echterdingen, Germany) was connected to control and to read out the integrated sensor module. The total height of the side member (with the mounted CFRP plate) in combination with the absorber was 0.17 m.

A displacement sensor as part of the test set-up measured the compression of the crash absorber over the progression of the collision. Additionally, a load cell at the drop tower recorded the force signal of the falling mass. The falling mass was 60 kg, falling height 2 m.

### 6.2. Results

The principle sensing behaviour of the self-sensing composite showed correct sensing. There was no significant measurement deviation by the sensor module before and after the integration into the CFRP structure. In addition, after the applied environmental loads to the self-sensing composite, all sensing parameters were within the tolerance range. The specification of the established automotive sensor defines the tolerances. The experiments are presented in detail in [61,74]. The following outlines the crash sensing behaviour of the self-sensing composite.

In the component test, the falling mass reproduces a collision of the component. By the present test set-up, the falling mass loaded the component and the absorber axially and stimulated the entire system. The crushing of the absorber corresponded to comparable component tests, with a formation of round lobes [69,72].

The objective of the experiment was the acceleration signal of the self-sensing composite over the progression of the collision. The examination focused on whether the signal crash typically followed the vibration response of the component. The basis of valuation was the force signal.

**Figure 10:**
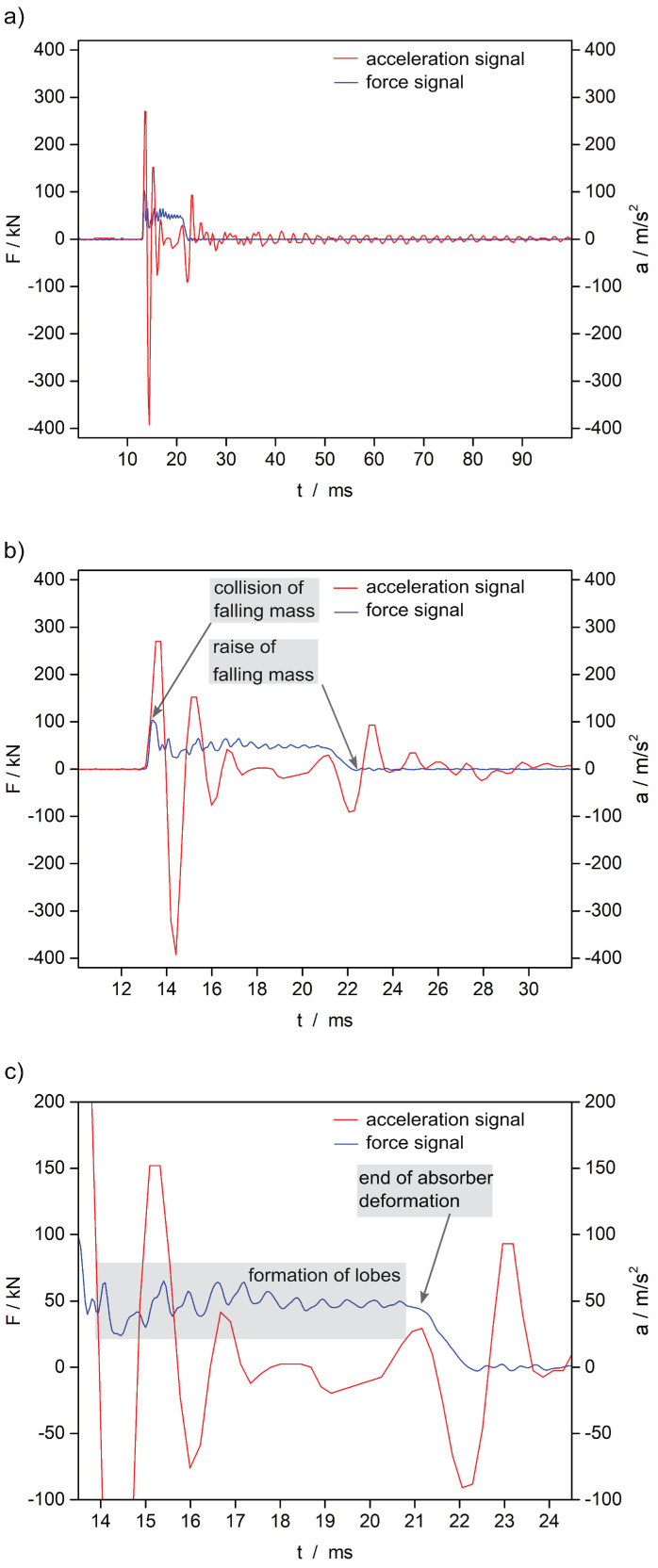
Signals during the component test: (**a**) complete signals, (**b**,**c**) detailed representations.

The force signal at the crash absorber and the acceleration signal of the self-sensing composite are plotted in Figure 10a. First of all, the force signal showed the typical system behaviour of the component during a collision. Qualitatively it was equivalent to the behaviour during real crash tests [69]. During the collision, a change in force is associated with structural stimulation. The corresponding propagation and reflection of the wave result in the measured acceleration. The effect is apparent in the moment of a force gradient. The details of the signals in Figure 10b,c demonstrate this in the experiment. The force and the acceleration signals both return the beginning of the collision at 13 ms (Figure 10b). At this point a steep rise of the force signal at the crash absorber occurred. At the same time, the acceleration signal of the self-sensing composite showed a positive amplitude. Thus, the self-sensing composite reacted to the stimulation of the system immediately. The entire system had been in equilibrium until the impact of the falling mass. Consequently, no forerunning oscillations were superimposed and the acceleration signal directly reflected the first wave of stimulation. As the collision developed, the absorber followed with the typical crushing. The arising oscillation of the force signal at a mean force of about 50 kN corresponded to the crushing (Figure 10c). The force progression at low frequency was plausible and material-typical for the aluminium crash absorber [69]. Without delay, the acceleration signal reflected the oscillation of the force by positive and negative amplitudes within the measuring accuracy. In the further process of the collision, the crushing of the absorber reduced the collision energy and the vibration. Therefore, the stimulation is no longer introduced completely into the system. As a consequence, the acceleration signal was dampened (Figure 10c). Between 20 ms and 21 ms, a spring back of the force occurred within milliseconds. A quick decrease in force was noticeable. The effect resulted once more in the stimulation of waves to which the acceleration signal reacted with a full oscillation (Figure 10c) starting with a negative amplitude due to the increase of force. At about 21 ms the force decreased gradually. The absorber deformation ended. Reaching 22 ms, the falling mass raised from the component and the force reached the zero level (Figure 10b). No further force impact occurred, whereby the system oscillation decreased. As a response, the oscillation of the acceleration is also reduced to signal noise.

As a whole, the comparison of the signals showed that the acceleration measured by the self-sensing composite correlated with the force progression at the crash absorber. The self-sensing composite plausibly reflected the system behaviour as a response to the stimulation. At the used position the sensor observed accelerations due to elastic deformation waves which were introduced in the side member by changes of the impact force.

### 6.3. Discussion

In the component test, the self-sensing composite was a fixed part of the component. It was stimulated during the collision. The acceleration signal of the self-sensing composite was compared to the force progression at the crash absorber. The behaviour of the two physical values was equivalent to system response. The acceleration signal clearly reflected the characteristic wave propagation of the stimulation. This takes into account that the acceleration of the system is merely a response to gradients of the force. The acceleration signal cannot directly be deduced from the force signal because the sensing system is not connected to the decelerated part (falling mass) of the test rig. It is connected to the fixed part of the test rig. Therefore, it measured the compression waves of the structure during the impact. Accordingly in the experiment the measured acceleration signal is not proportional to the force signal. Further, the acceleration signal is composed of the acceleration and the stimulation of the system. A numerical analysis of the two signals requires a comprehensive description of the entire system. Models to describe the complexity with all influencing parameters (material, geometry etc.) were elaborated [69,75,76]. The models are another focus of research and go beyond the present scope.

The conclusion is that the typical functional behaviour of the self-sensing composite is present in the event of a collision. It corresponds to the sensing behaviour of comparable acceleration sensors, which are specifically designed for crash tests [69]. Therefore, the integration of the automotive acceleration sensor in an FRP structure does not restrict its use for crash sensing. The thesis applies under the premise that the present observation was phenomenological. A differentiated evaluation of the sensing behaviour presupposes an analysis by the above-mentioned models. Likewise, a profound statement on the sensing behaviour of the self-sensing composite as part of a complete vehicle cannot be given. The vehicle type and the mounting site of the sensor at or inside the vehicle structure determine the parameters of the transmission function. However, the present results give a functional estimation of the self-sensing composite during a collision.

## 7. Conclusions

As a conclusion of the research findings, self-sensing FRP composites are suitable for vehicle structures with reasonable efforts. Integration of (automotive) sensors as a serial product in FRP structures of good quality was demonstrated. Structural optimization of future self-sensing composites is likely possible by an adjusted design. The thinning of the sensor device is an appropriate future measure. A smaller sensor module should prospectively replace the sensor module of the current automotive acceleration sensor. In other products such as mobile phones, pedometers or motion capture wearables, acceleration sensors of small dimensions are already state of the art. In addition, microsensors have already been used to measure acceleration for different applications [77,78]. According to the state of research, accelerometers are available even as nanoelectromechanical systems [79]. Key drivers for the development of small and cost-effective sensors are primarily in the context of IoT and industry 4.0 [80,81]. In the near future, commercially available and sufficiently validated sensors with small dimensions should also be available for automotive applications. Furthermore, the efficient integration of completely film-based multi-sensor platforms [82] is a future step, including supply units, electronics for data processing and antenna connection once they are commercially available.

From a functional point of view, this paper outlines the requirements for the crash sensing of the integrated sensor as a primary feature. The experiments showed that the typical functional behaviour of the self-sensing composite is present in the event of a collision. A comprehensive investigation of the integrated sensor inside a complete vehicle structure is the next appropriate step. Thereby, it is mandatory to describe the entire system’s behaviour of the vehicle structure. Because of the complexity, physical analogous models might be required. Additionally, analytical methods to describe the deformation processes of crash structures are helpful [70]. The behaviour of FRP structures during a collision differs from established metallic vehicles and is a broad field of employment. In addition, to determine the parameters of the sensor’s transmission function for different types of structures, forecasting methods are available [69].

The integration approach is also transferable to products that go beyond the automotive sector. Apart from e.g., a rocker panel, an underbody or a battery housing of a vehicle, sports equipment, machines and containers were taken into account [51,56]. Thereby, the integration approach is not limited to FRP structures. It is applicable to polymer products in general e.g., in the context of connectivity and IoT, industry 4.0, entertainment, smart home or everyday management. Established sensors can be integrated into household appliances, helmets or crash test dummies. The approach is particularly efficient if functional safety is paramount in the sensor availability. This involves for example the autonomous driving or autonomous robots [83].

Overall, the present approach redefines the sensor packaging: the product becomes the new housing of a sensor; moreover it becomes a part of the sensor. Thus, the product influences the sensing behaviour; vice versa, the integrated sensor influences the (structural) behaviour of the product. The combination of the two actually independent systems—product and sensor—to an entire system opens up new potentials. However, a conceptual rethinking is required. The present work provides a starting point.

## Figures and Tables

**Table 1:** Specific mechanical values of the CFRP composites (standard deviation).

Specific Value	Reference		Self-Sensing		Regular
CFRP structure [0/90]_S_					
**Tensile test (DIN EN ISO 527-4)**					
Fibre content (%_vol._)	60		36		35
Tensile modulus (GPa)	69.2 (2.1)		43 (1.2)		41.4 (1.3)
Tensile strength (MPa)	1064 (98.3)		699 (19.0)	*	607 (48.5)
Elongation at break (%)	1.47 (0.05)		1.54 (0.03)	*	1.3 (0.05)
**Compression test (DIN EN ISO 14126)**					
Fibre content (%_vol._)	62		35		35
Compressive modulus (GPa)	69.3 (1.0)	**	41.4 (0.2)		40.1 (1.5)
Compressive strength (MPa)	572 (22.2)	**	283 (37.2)	*	395 (12.3)
Compression (%)	0.92 (0.04)		0.71 (0.11)	*	1.15 (0.06)
**Bending test (DIN EN ISO 14125)**					
Fibre content (%_vol._)	61		35		35
Bending modulus (GPa)	75.3 (3.1)		46.6 (2.5)		44.5 (2.2)
Bending strength (MPa)	918 (68.2)		683 (28)		663 (40.9)
Bending strain (%)	1.33 (0.1)		1.74 (0.1)		1.76 (0.06)
CFRP structures [±45]_S_					
**Tensile test (DIN EN ISO 527-4)**					
Fibre content (%_vol._)	61		35		34
Tensile modulus (GPa)	4.6 (0.04)		2.9 (0.03)		2.7 (0.2)
Tensile strength (MPa)	62.8 (0.3)		51.4 (0.8)		50.2 (1.3)
**Compression test (DIN EN ISO 14126)**					
Fibre content (%_vol._)	60		33		33
Compressive modulus (GPa)	15.2 (1.0)		10.6 (0.8)	*	8.3 (0.5)
Compressive strength (MPa)	160.1 (9.4)	**	120 (1.2)		122 (0.6)
Compression (%)	10.27 (0.51)		10.87 (0.34)	*	12.72 (0.02)

## Abbreviations

The following abbreviations are used in this manuscript:

CFRP	Carbon fibre-reinforced polymers
CNT	Carbon nano tubes
CT	Computer tomography
FRP	Fibre-reinforced polymers
IoT	Internet of Things
LCM	Liquid composite moulding
MEMS	Micro electro mechanical system
RTM	Resin transfer moulding
